# FIB the fractured femur

**DOI:** 10.1186/cc12201

**Published:** 2013-03-19

**Authors:** H Shahzad, M Majeed, D Yeo, V Gupta, U Salanke

**Affiliations:** 1 University Hospital, Birmingham, UK

## Introduction

The lumbar plexus block provides excellent analgesia after hip and knee surgery. One approach to the three major nerves (femoral, lateral cutaneous and obturator) of the lubar plexus is the fascia iliaca compartment block, first described by Dalens and colleagues. Using this blind approach, complete nerve block in the distribution of the lumbar plexus may be achieved only in 38% of cases. Ultrasound-guided regional techniques offer a number of advantages including real-time needle guidance and direct observation of local anesthetic spread within tissue planes.

## Methods

We hypothesized that real-time UFIB could be successfully performed in the ED and would provide an excellent adjunct or alternative to repeat doses of IVMS for pain control in patients with HFx. The study was conducted at University Hospital, Birmingham, where we see about 90,000 patients every year. All patients with confirmed femoral (neck/shaft) fractures and pain score >7 were included in the study. Patients with local wounds, or suspected significant pelvic injury were excluded. A combination of 15 ml lignocaine 1% and 15 ml bupvivcaine 0.25% was used. VAS (0 to 10), pain on movement of the leg and patient satisfaction were used to assess the outcome. The assessments were made before the FIB, at 15 minutes, 30 minutes and 45 minutes.

## Results

A convenience sample of 19 patients was enrolled in this study. The compartment block was placed by three mergence ultrasound trainers. The mean age was 58.5 years. There were 13 females and six males. The mean pain score was 9.5 at time 0. The mean pain score had improved to 5.5 at 15 minutes, 4.5 at 30 minutes and 3 at 45 minutes, respectively. The patient satisfaction was scored 4.5 on a scale of 1 to 5 where 5 was the most satisfied. No patient required any further analgesia up to 90 minutes and no issues were raised about the pain or discomfort upon patient transfer. We have no documented complications or side effects.

## Conclusion

FIB is now widely used by non-anaesthetic trainees to combat pain in preoperative care due to its safe landmarks. Our results have shown that it can be used safely and effectively for pain management in hip fracture by emergency physicians, who are trained in the technique, on the shop floor.

